# The effect of background music on stress in the operating surgeon: scoping review

**DOI:** 10.1093/bjsopen/zrac112

**Published:** 2022-10-12

**Authors:** Anantha Narayanan, Lydia Pearson, James P Fisher, Manar Khashram

**Affiliations:** Department of Surgery, University of Auckland, Auckland, New Zealand; Department of Vascular Surgery and Endovascular Surgery, Waikato Hospital, Hamilton, New Zealand; Department of Vascular Surgery and Endovascular Surgery, Waikato Hospital, Hamilton, New Zealand; Department of Physiology, Faculty of Medicine and Health Sciences, University of Auckland, Auckland, New Zealand; Department of Surgery, University of Auckland, Auckland, New Zealand; Department of Vascular Surgery and Endovascular Surgery, Waikato Hospital, Hamilton, New Zealand

## Abstract

**Background:**

Despite the ubiquitous sounds of music playing in operating theatres (OTs) around the world, the effect that music has on intraoperative clinician stress is ill-defined. In the present scoping review the aim was to map the available evidence for the effect of background music in the OT on the experience of stress in the operating surgeon.

**Methods:**

The present review was conducted in accordance with the PRISMA Protocols for Scoping Reviews. Using Embase, MEDLINE, and the Cochrane databases, peer-reviewed research studies reporting the effect of intraoperative background music on an outcome measure of clinician stress or respondent perceptions on this effect were included.

**Results:**

A total of 4342 studies were screened and 15 studies met the inclusion criteria, of which 10 were interventional studies, and five were observational survey-based studies. Of the 10 interventional studies, four showed reduced anxiety and mental workload scores with music, although only two demonstrated a significant improvement in a physiological outcome. The survey-based studies generally reported a positive perception among surgeons and theatre staff towards the effect of music on OT stress.

**Conclusion:**

While there is generally a positive perception towards intraoperative music and surgeon stress, there are few objective physiological and psychological data to support this. Studies were varied in their design. The present review can be used to guide future experimental, observational, and mixed-method research on this topic.

## Teaser

Music is played widely in operating theatres throughout the world; however, the objective effect it can have on a surgeon’s experience of stress is not well understood. This scoping review maps the present literature in this field and proposes future avenues for research.

## Introduction

Surgery can be a stressful exercise that warrants expert execution of both technical and non-technical skills (such as communication, teamwork, and rapid decision-making) under pressure. The feeling of stress, and managing its flow-on effects to performance, is one that unites all surgeons in their experience^1^. Music can be one way in which surgeons can alter their operating environment, and it is perhaps not surprising to find that it is played commonly in operating theatres (OTs) throughout the world^2–4^. Though commonplace, there is disagreement in the literature regarding the perceptions of the benefits or harms that music can have in this context.

While it is clear that noise is deleterious to the surgical team^5–10^, many clinicians find music to be a generally favourable part of the theatre environment^4,11,12^, with music being seen as improving calmness^2^, stress^13,14^, mood, and surgeon and overall team performance^4^. However, respondents’ opinions differed when it came to the distracting effect of music^11,13^, particularly at times of critical situations^2–4,11^—factors that may affect performance, increase the feeling of difficulty, or stress^15^. Communication is another contentious area, where several studies report no effect or a positive influence with music^2,4,14,16^, though others found a reduction in auditory speech perception^10,17^ and an increase in repeated request rate^18^.

Since the seminal paper by Rauscher *et al.* on the ‘Mozart effect’, demonstrating an improvement in spatial reasoning skills after listening to Mozart, there has been interest in the interaction between music and task performance^19^. Recent systematic reviews concluded that having background music may also improve surgical accuracy and speed^20^, and reduce mental workload in the simulated setting^21^. It could be that the experience of stress may be a contributing or even fundamental factor bridging the gap between music and surgical performance.

In fields such as music therapy and occupational ergonomics, music is used to facilitate mood regulation^22^ and to adjunct learning^23^; however, there is a dearth of objective and experimental data in the literature assessing how background music affects a surgeon’s stress and anxiety levels intraoperatively, and how this applies to surgical performance. For these reasons, the PRISMA Extension for Scoping Reviews (PRISMA-ScR) guidelines^24^ were followed to conduct a systematic review of the relevant literature regarding the effect of background music on a surgeon’s intraoperative experience of stress and anxiety, with the aim of providing a platform with which to inform future research.

### Methodology

The study protocol was developed using the PRISMA-ScR with the aim of answering the research question: ‘For surgeons who are operating, is background music, when compared with noise or silence, effective in reducing the experience of intraoperative stress?’ To identify potentially relevant papers, Ovid MEDLINE, Embase, and Cochrane Reviews (including the Trial Registry) were searched. The search was performed by the research team in conjunction with a university librarian (see Appendix S1 for search terms). The final search results were exported into EndNote and the duplicates were removed.

Papers were included in the present review if they

- involved background music in relation to surgeons, surgical environments, or the performance of surgical tasks.
- measured or assessed a specific dimension of physiological or psychological stress, anxiety, mental workload or task-load of the surgeon or task performer.
- surveyed clinician perceptions of the above.

Further inclusion criteria included peer-reviewed journal papers, written in English that involved human participants and described at least one measure of stress or reported perceptions of stress. Quantitative, qualitative, and mixed-method studies were included to consider different aspects of measuring stress or anxiety. Papers were excluded if they did not fit into the conceptual framework of the study—if the outcome was only surgical task performance or efficiency, related to OT noise without reference to music, or solely focussed on the effect of music on patients. Authors of conference abstracts were contacted for pre-prints, but if there was no publication available or no response, these were excluded.

Using Rayyan systematic review software^25^, titles and abstracts were sequentially screened by two reviewers (A.N. and L.P.) for relevant publications, after which full-texts were collected and assessed for eligibility to be included. When disagreements on study selection and data extraction arose, consensus was sought from a third-party investigator (M.K.). Manual examination of reference lists of included studies to find relevant studies was performed additionally. Where a related systematic review was identified, papers that met the inclusion criteria and had not been included in the initial database search were reviewed and assessed for inclusion.

### Risk of bias assessment

The present review was not aimed to synthesize data about intervention effectiveness; thus, an assessment of the risk of bias was not performed.

### Data-charting process

A standardized data-charting form was jointly developed by the research team in an iterative process to determine which variables to extract. The eligible studies were examined, and relevant information was captured on study characteristics by two reviewers independently (A.N. and L.P.). Disagreements were resolved through discussions between the two reviewers or referred for adjudication by a third-party investigator (M.K.).

### Data items and synthesis

Studies were grouped by study design and data were extracted on article characteristics (for example, year of publication, country of origin, type, and number of participants). For the interventional studies, study design, the nature of the intervention, the surgical task, measure of stress, and main findings were extracted. Data on the intervention included the number of arms in the trial, type of music and how it was played, and definition of the control group (control music, pre-recorded OT noise, or silence). Physiological measures of stress such as heart rate (HR), blood pressure (BP), heart rate variability (HRV), and other measurements of autonomic phenomena were recorded. Psychological instruments (questionnaires) that were used were recorded. For survey-based studies, the questions pertaining to an experience of stress were extracted and the relevant findings were recorded.

## Results

### Characteristics of included studies

**Fig. 1:**
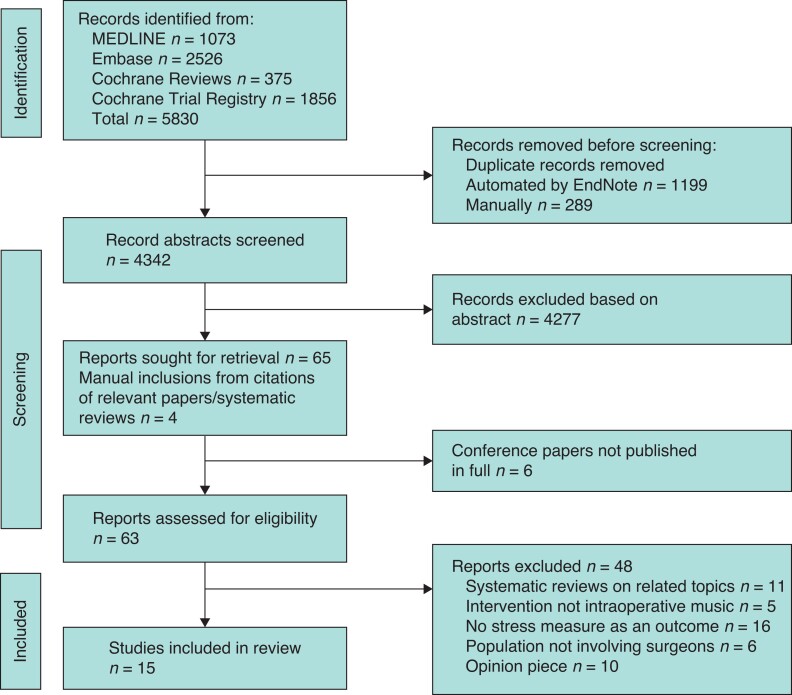
PRISMA flow diagram for included/excluded papers

A total of 5830 citations were identified. After de-duplication, 4342 unique citations were identified, and their abstracts were screened for relevance, yielding 65 reports that were sought for retrieval. Abstracts that the two primary reviewers (A.N. and L.P.) had conflict on were included. Of the 65 reports, six were conference abstracts. Primary authors were contacted through academic social networks or identifiable corresponding emails for completed or pre-printed manuscripts, but these yielded no further inclusions. The citations of retrieved systematic reviews were also screened, and four further papers were included for retrieval. The full text of 63 papers were then assessed, with six conflicts adjudicated by the third reviewer (M.K.). In total, 15 papers were finally included in the scoping review (*Fig. 1*). Ten papers were interventional studies, and five were prospective survey-based cross-sectional studies.

### Interventional studies

#### Trial design

There were 10 interventional studies carried out across Thailand, USA, France, The Netherlands, China, Switzerland, and India with one being a multicentre study (*Table 1*). Three studies involved fully trained surgeons^26,27,34^, one involved inexperienced junior surgeons^29^, five studies involved medical students as participants^21,28,30,31^, and one study included both residents and students^33^. Only one study was carried out in a clinical operating environment (non-simulated), and this also included patients in the study design^26^. The predominant trial design was a two- or three-arm crossover randomized clinical trial (RCT) with a surgical simulator (five trials)^21,28,29,31,32^, but included were three non-surgical skill-based RCTs^27,33,34^, one RCT in a clinical setting^26^, and one anatomy dissection classroom RCT^30^.

**Table 1:** Summary of interventional studies examining the effect of music on a measure of stress

Interventional studies									
First author (country), and year	Study design, and number of arms	Environment	Study population (*n*)	Intervention	Control	Task	Stress outcome measures	Other outcome measures	Main findings relating to effect of music on stress
**Alam *et al.* (Thailand) 2016** ^26^	RCT, 3	Procedure room	Patients and surgeons (155)	Guided imagery *versus* relaxing music	No music	Skin lesion excision under LA	STAI-6	Patient’s STAI-6, visual-analogue pain scale, BP and HR	Surgeon anxiety was significantly lower when operating on patients in the guided imagery and relaxing music groups.
**Allen *et al.* (USA) 1994^27^**	RCT, 3	Lab	Male surgeons (50)	Self-selected *versus* investigator-selected (Pachelbel's Canon in D) music conditions.	No music	Serial subtraction (arithmetic tasks)	Skin conductance, HR (from cuff), BP (non-invasive cuff)	Task speed and accuracy	Autonomic reactivity for all physiological measures was significantly less in the surgeon-selected music condition (speed and accuracy were better) than in the experimenter-selected music condition, which in turn was significantly less than in the no-music control condition.
**Gao *et al.* (China) 2018^28^**	RCT, 3	Surgical Simulator	Postgraduate medical students familiar or experienced with surgical skills (24)	OR noise *versus* music (Beethoven’s Moonlight Sonata)	No music	Laparoscopic appendicectomy on the MIS-Laparo Virtual Simulator	NASA-TLX, eye movement tracking and pupil size	Task completion time, motion analysis	Significant pupil dilatation, higher mental workload and worsened performance, with OT noise and silence, but no difference between music and silence.
**Miskovic *et al.* (Switzerland) 2008^29^**	RCT, 3	Surgical Simulator	Junior surgeons with no previous laparoscopic experience (47)	‘Activating’ music *versus* ‘deactivating’ music	No music	Laparoscopic cholecystectomy simulation via the Clip and Cut module of the Xitact LC 3.0 virtual reality simulator	Mean HR and HRV (device not stated)	Task completion time, global/error performance score, instrument travelling distance	No significant findings between groups on autonomic or performance parameters. Non-significant trend towards impaired performance with activating music.
**Bellier *et al.* (France) 2019^30^**	Cluster RCT, 2	Anatomy lab/classroom	Second year medical students (187)	Instrumental background music	No music	Anatomy lab cadaver dissection	STAI (full version in French)	Grading of practical work by assessors, student-reported satisfaction	A significant relative decrease in acute anxiety, was found for the music intervention group. Music also had a positive impact on performance with students in the intervention group attaining higher grades than those in the control group.
**Fu *et al.* (Netherlands) 2020^21^**	Crossover RCT, 2	Surgical Simulator	Medical students who were novices to laparoscopy (107)	Self-selected music	Recorded operation room noise	Validated, custom-made laparoscopic box simulator using the peg transfer task	SURG-TLX, HRV (chest strap)	Task completion time, motion analysis	Music significantly decreased mental workload, reflected by a lower score of the total weighted Surgery Task-Load Index in all but one of the six workload dimensions. Music did not significantly improve laparoscopic task performance.
**Oomens *et al.* (Netherlands) 2020^31^**	Crossover RCT, 2	Surgical simulator	Medical students (60)	Self-selected music	No music	Peg transfer task on a laparoscopic box trainer	HR, BP (non-invasive cuff), SURG-TLX	Task completion time, instrument path length, normalized jerk	In the music condition: mental workload was significantly reduced, but no difference in HR or BP. Overall faster performance and more efficient path length.
**Pluyter *et al.* (Netherlands) 2010^32^**	Crossover RCT, 2	Surgical simulator	Medical interns with no laparoscopic experience (12)	Social–technological distracting conditions: standardized combination of music (2 popular songs) mixed in parallel with 30 s of case-irrelevant communication. (also had factor of non-optimal camera control *versus* optimal)	No distraction/no music	Laparoscopic cholecystectomy simulation via the Clip and Cut module of the Xitact LC 3.0 virtual reality simulator	HR, BP (non-invasive cuff)	Task completion time, objective performance score recorded by the simulator, perceived irritation	No difference in physiological parameters. Under distracting conditions, there was a significant decline in task performance and significantly increased levels of irritation.
**Rogers *et al.* (USA) 2019^33^**	Crossover RCT, 3	Lab	10 undergraduate students, 22 medical students, and 5 neurosurgical residents (37)	Pre-recorded sounds of a loud operating room *versus* self-selected music	No music	Non-surgical fine motor dexterity tasks (MLS motor performance series of the Vienna test series by Schuhfried), battery of cognitive thinking tasks	Profile of mood states	Task performance scores	Self-selected music resulted in a significant decrease in feelings: anger, hostility, confusion, bewilderment, fatigue, inertia, tension, and anxiety and a significant improvement in total mood disturbance. Music improved the speed and precision of movements and information processing skills and improved complex attention and mental flexibility
**Mitta *et al.* (India) 2019^34^**	Single-arm interventional trial, 5	Lab	Surgeons of varying specialties (45)	5 music conditions including: ambient OR noise, music of personal preference, western classical, heavy metal and pop music	No Control	5 different types of non-surgical tasks: trail marking, jigsaw puzzle, backward counting, comprehension and memory game using cards	Mean HR and MAP (device not stated)	Task performance	There was no significant increase in the pulse rate and MAP across various time points between different genres of music playing except for MAP between baseline and music of choice. Familiar music improved performance with memory tasks, but impaired others.

STAI, State-Trait Anxiety Inventory; STAI-6, abbreviated form of STAI; LA, local anaesthesia; BP, blood pressure; HR, heart rate; RCT, randomized clinical trial; NASA-TLX, precursor to Surgical Task-Load Index; OT, operating theatre; OR, operating room; HRV, heart rate variability; MLS, motor performance series; MAP, mean arterial pressure.

#### Control and music conditions

There was variation in the control condition, with some studies being silent, and others having recorded ambient OT noise. In five studies, investigators/authors used participant-selected music^21,27,31,33,34^. Other examples of music conditions included ‘relaxing’ music (a slow tempo of soothing music and nature)^26^, ‘activating or de-activating music’ (chosen to evoke a mental condition for example Wagner’s *Valkyrie* or certain tracks by Nicholas Gunn for example, *Seeking Serenity*)^29^, classical music such as Pachelbel’s *Canon in D*^27^ and Beethoven’s *Moonlight Sonata*^28^, or a wide selection of genres^34^.

#### Psychological outcome measures

A psychological measure was recorded in six studies, of which three different psychological instruments were used. The Surgical Task-Load Index (SURG-TLX) (and its precursor the NASA-TLX)^35^, a 20-point, surgical specific, multi-dimensional rating scale that has six bipolar dimensions, was used in three studies. Two studies^21,31^ found a reduction in mental workload with music when compared with a non-music group, although Gao *et al.* (where the NASA-TLX was used) found no difference^28^.

The State-Trait Anxiety Inventory (STAI) is a widely used and established psychological instrument that is used to evaluate the current state of anxiety and stable aspects of anxiety proneness^36^. This inventory, its abbreviated and validated^37^ (STAI-6) and full-French format, was used in two studies^26,30^. Alam *et al.* found lower STAI-6 scores (less anxiety) in surgeons when having relaxing intraoperative music played during local anaesthetic skin lesion operations^26^, a finding replicated in medical students in the anatomy laboratory more recently by Bellier *et al.*^30^.

The Profile of Mood States is a self-reported 65-item inventory to assess transient and enduring mood changes and a commonly used tool to measure psychological distress across several ‘mood categories’^38^. It was used only in one study. Rogers *et al.* found that in comparison with silence, having music of the subject’s choosing resulted in a significant decrease in feelings of anger and hostility, confusion and bewilderment, fatigue and inertia, and tension and anxiety—with an improvement in total mood disturbance^33^.

#### Physiological outcome measures

A physiological measure was recorded in eight out of the 10 studies, with a broad range of parameters measured, and a variety of collection devices. In six studies, HR was measured^21,27,29,31,32,34^ and two of which also calculated HRV^21,29^. HRV (the fluctuation of the R-R interval) has in recent decades become a popular clinical and research tool to assess autonomic nervous system function due in part because of its ease of access and non-invasive nature^39^. HR was measured with a BP cuff in two studies^27,31,32^, a chest strap^21^ and photoplethysmography^27^ in one study each, and the remaining two studies did not state the collection device used. Systolic and diastolic BP were measured in four studies^27,31,32,34^, and one study used a calculated mean arterial pressure^34^. Skin conductance^27^ and eye movement/pupil size^28^ were used in one study each. Allen *et al.*, when challenging surgeons with arithmetic tasks, observed a significantly lower HR, BP, and skin conductance response frequency (indicating lower autonomic reactivity) in the ‘familiar music’ condition when compared with the ‘Pachelbel’s canon’ condition or with no music^27^. Gao *et al.* reported a significant difference in pupil dilatation during simulated laparoscopic cases with noise when compared with the control (silence), though no change in the music arm was seen^28^. No other studies found statistically significant differences in physiological parameters when comparing music and non-music trial arms.

#### Other findings

Nine of the 10 studies measured task performance (either speed, accuracy, or efficiency) as an outcome, with three of these studies reporting a statistically significant improvement in task performance in the music arms^21,27,31^.

#### Survey-based studies

There was a total of 924 healthcare providers surveyed in these five studies (*Table 2*). There were between 274–355 surgeons and surgical residents surveyed (a range is provided as there is no explicit number in Ullman *et al.*)^2^. The effect of music on stress or related moods and emotions often made up a small part of these surveys, but focussed on questions relating to calmness, vigilance, focus, concentration, autonomic reactivity, and reduction of stress. While communication was also a common theme, this factor was outside the scoping review.

**Table 2:** Prospective survey-based cross-sectional studies

First author (country), and year	Sample size	Types of participants	Questions relating to stress	Main findings relating to effect of music on stress
**George *et al.* (India) 2011^14^**	100	Randomly selected surgeons (44), anaesthesiologists (25), and nurses (31)	Do you think music improves concentration? Do you think it reduces your autonomic reactivity in stressful surgeries?	63% agreed that playing music improved their concentration and 59% of the respondents thought that music helped in reducing their autonomic reactivity in stressful surgeries.
**Makama *et al.* (Nigeria) 2010^13^**	162	All theatre staff. 27 (16.7%) surgeons, 21 (13.0%) obstetric and gynaecologist, 17 (10.5%) ophthalmologists, 14 (8.6%) maxillofacial surgeons, 15 (9.3%) orthopaedic surgeons, 18 (11.1%) anaesthetists, 22 (13.6%) nurses, 17 (10.5%) theatre attendants, 11 (6.8%) others including patients	Does the respondent have knowledge of the effect of music in terms of anxiolytic effect, minimization of annoyance reduction of stress and whether familiarity of music has an enhanced effect.	The proportion of respondents with knowledge of therapeutic efficacy of music in the theatre revealed Anxiolytic (93%) Reduced of stress (91.4%) Minimizes annoyance (79.6%) Familiarity of music played enhanced performance (86.4%)
**Narayanan *et al.* (New Zealand) 2018^4^**	101	All theatre staff. Surgeons (37%), anaesthetist (29%), nurse (25%), anaesthetic technician (10%)	Perceptions of music on own calmness, own vigilance, own focus, mood in theatre	Ignoring those responding that music had no effect, music was seen to improve calmness (84%), and mood (97%). There was no significant evidence of a direction of effect for vigilance or own focus. Overall worsened sense of own communication
**Ullman *et al.* (Israel) 2006^2^**	171	Senior physicians (72) and residents (36), comprising anaesthetists and other surgical specialties, nurses (63)	‘During surgery, music makes me calmer’ ‘Do you think that music in the OR affects the communication between staff?’	Music made most feel calmer (65.8%), and positively influenced communications (63%)
**Yamasaki *et al.* (USA and Japan) 2016^16^**	672	282 patients and 390 providers. Providers: 39 attending surgeons, 60 surgery residents, 52 attending anaesthesiologists, 45 anaesthesiology residents, 172 OR surgical nurses and 22 anaesthetic nurses	‘How does music impact your concentration?’	Nurses held the most positive views of music’s impact on concentration in the OT. Surgery providers tended to have a more positive view than did anaesthesiology providers regarding the impact of music on concentration in the OR

OR, operating room; OT, operating theatre.

All the papers included in this review reported positive findings with regard to perceptions of intraoperative stress and concentration with background music. Narayanan *et al*. found that most respondents felt that music improved calmness and mood^4^, a finding echoed by Ullman *et al.*^2^ and Makama *et al.*^13^. George *et al.* found that most respondents thought that music helped to reduce their ‘autonomic reactivity’ in stressful surgeries^14^. A majority of respondents of George *et al.* and Yamasaki *et al.* felt that music improved concentration in the OT^14,16^, but this was equivocal with regard to vigilance or focus in the New Zealand study^4^.

## Discussion

The present study represents the first attempt to comprehensively review the literature of the actual and perceived effect that music has on a surgeon’s experience of stress while operating.

Stress is recognized as an important performance-shaping factor in the domains of aviation^40^, the military^41^, and elite sports^42^, by distracting attention from the primary task, disrupting the ability to use working memory, and by limiting the opportunity to gather enough information to make a decision^43^. The Yerkes Dodson Law, suggests an empirical relationship between arousal and performance, dictating that although a certain degree of stress can facilitate the performance of complex or difficult tasks, when levels of arousal become too high, performance decreases^44^—a relationship often represented graphically as a bell-shaped curve^45^. A systematic review by Arora *et al.* in 2010 comprising 22 studies showed a heterogenous array of measures of stress used, and ultimately concluded that ‘within surgery, a distinct lack of performance data precludes evidence-based conclusions to be drawn on the effects of stress or the mechanisms involved’^46^. Music has for centuries been used in all aspects of life to modulate mood, emotions, and generate physical and physiological responses^47,48^, hence it is not surprising that its use has become established within OTs around the world.

It could be that the level of stress that a surgeon experiences may be a contributing element bridging the gap between music and surgical performance. Music has long been used intentionally by listeners to regulate their moods and emotions^49^, with strong support for the notion that music can generate physiological, arousal or relaxative responses^50^. Since Rauscher’s seminal paper in 1993 describing the beneficial ‘Mozart Effect’ of music on spatial reasoning tasks^19^, a number of studies have tested this effect in surgery. A recent systematic review ultimately concluded that classical music at a low to medium volume could improve accuracy and speed, though cautioned taking into consideration its distracting effect^20^. The survey-based studies in this review demonstrate a general perception of theatre staff to the positive impact on music towards mood and stress; however, within the OT environment it can still be divisive. A survey of anaesthetists in Glasgow found that while music was largely liked, a proportion felt that music reduced their vigilance, and was distracting when a problem was encountered during surgery^3^. Widely used in OTs around the globe, there is also a body of evidence to support the benefit that music has to the patient, in that it has been shown to have anxiolytic effects ^51,52^, can improve pain control^53,54^, and even reduce anaesthetic or pre-medication requirements^55,56^.

A number of studies included in this review used OT noise as a control when compared with music, with one even using music as part of a social–technological distracting condition^32^. The effect of general noise on the theatre environment has been studied and it has been clearly shown to have a negative impact on surgical team performance^5–10^ and patient safety^57^; however, the differentiation between noise sources and music has not been clearly ascertained and could depend on the situation and the people within the environment^57^.

The interventional studies in this review apply a heterogenous mix of physiological and psychological measures in attempting to capture the experience of stress. There were some notable results with the STAI and SURG-TLX, which are useful and validated tools to assess workload and anxiety^35,36^. Stress is well known to be driven by physiological responses mediated by the autonomic nervous system, in a fine balance between the sympathetic and parasympathetic levers. Within this review, there were no notable outcomes with physiological measures, and it is hypothesized that if very small differences in BP or HR exist between music and non-music conditions, a very large number of participants in a controlled experimental trial are needed. HRV is becoming one of the most widely used measures in the field of psychophysiology for indexing cardiac vagal tone in response to various stimuli^58^, in part due to more accessible technology and the apparent ease and non-invasiveness of data derivation—though only two papers were found that utilized this measure, although neither found a significant difference between control and interventional groups^21,29^.

Nine out of 10 of the interventional studies were carried out in non-operating environments and four involved trained surgeons. From a pragmatic point of view this is understandable; however, it means that the findings are not easily generalizable to the surgeon in the operating environment. When performing interventional studies with physiological monitoring devices, care is needed when approaching a clinical environment to ensure that a study is carried out in a non-interrupting fashion using equipment that is non-obstructive.

Overall, the results of the present scoping review suggest that while there is a widespread perception of the positive influence of music on stress in surgery, there is considerable heterogeneity in study design, and a lack of quality physiological data to corroborate this view with the real-world experience.

The data from this systematic review have been collated in a structured manner and are summarized to generate a point of departure for further and more robust survey-based, interventional and mixed-method research; however, given the vast array of study methodologies, study sizes, and measurement outcomes, it is challenging to draw a meaningful overall conclusion from the included studies. Furthermore, as this was a scoping review, study quality or risk of bias was not assessed, further weakening the ability to draw conclusions. As surveys are voluntary, the survey-based studies may preferentially include participants who have an interest or inclination to intraoperative music, and so the reported perceptions towards its effect may be skewed positively. Conference abstracts would comprise grey literature, and although authors were contacted for manuscripts, none of these was included in this study, resulting in the exclusion of these non-peer-reviewed research works.

Ensuring success and excellence in surgery involves maintaining and improving quality at the clinician, team, and organization level. Stress is a major contributor to surgical performance and so exploring ways to mitigate the stress response in an already stressful environment is important. Music is played commonly in theatres and is liked by many, though while interest in its effect is widespread, its true effect is not well known. The results from the present review have demonstrated the variability in studies designed with the goal of answering this question. While there is generally a positive perception towards intraoperative music and surgeon stress, there are few definitive objective physiological and psychological data to support this. The present review could be used as a starting point to guide future research, especially interventional studies, on this topic.

## Supplementary Material

zrac112_Supplementary_Data

## Data Availability

All data generated or analysed during this study are available on the medical literature databases Ovid MEDLINE, Embase, and Cochrane Reviews (including the Trial Registry). All papers included in this published article are listed in the references section. Data sets are available from the corresponding author on reasonable request.
