# Supplementary material for: The effect of background music on stress in the operating surgeon: scoping review
Source: BJS Open. 2022 Oct 12;6(5):zrac112. doi: 10.1093/bjsopen/zrac112 (PMC9553852; doi:10.1093/bjsopen/zrac112)
Supplement: zrac112_Supplementary_Data [file zrac112_supplementary_data.docx]

Embase <1980 to 2021 August 11>

1 music therapy/ or music/ 24553

2 auditory stimulation/ 36095

3 (playlist* or spotify or mp3 or ipod or music* or acoustic stimul* or auditory stimul* or song* or melod*).ab,ti,kw. 55240

4 1 or 2 or 3 85691

5 surgeon/ or surgery/ or medical procedures/ 691523

6 operating room personnel/ or operating room/ 47092

7 surgical stress/ 3369

8 anesthesia/ or anesthesist/ or anesthesiologist/ or anesthesiology/ 132590

9 (((operating or operation) adj3 (room* or staff or theatre*)) or surgeon* or an?esthe* or pre?operat* or post?operat* or peri?operat* or intra?operat* or surger* or surgic*).ab,ti,kw. 3335184

10 5 or 6 or 7 or 8 or 9 3480187

11 stress/ or psychophysiology/ or acute stress/ or behavioral stress/ or burnout/ or chronic stress/ or critical incident stress/ or emotional stress/ or job stress/ or life stress/ or surgical stress/ 231838

12 (stress* or perform* or demand* or focus* or anxiet* or relax* or work?load or task?load or heart rate or blood pressure or skin conduct* or STAI* or SURG?TLX*).ab,ti,kw. 8319191

13 11 or 12 8368921

14 4 and 10 and 13 2526

Ovid MEDLINE(R) Epub Ahead of Print, In Process & Other Non-Indexed Citations, Ovid MEDLINE (R) Daily, and Ovid MEDLINE (R) 1946-Present

1 Music/ 14910

2 acoustic stimulation/ or music therapy/ 48464

3 (playlist* or spotify or mp3 or ipod or music or radio or acoustic stimul* or noise or auditory stimul* or sound* or song* or melod* or tune).ab,ti,kw. 338863

4 exp Specialties, Surgical/ 206957

5 anesthesiologists/ or surgeons/ or neurosurgeons/ or orthopedic surgeons/ 12668

6 (((operating or operation) adj3 (room* or staff or theatre*)) or surgeon* or an?esthe* or pre?operat* or post?operat* or peri?operat* or intra?operat* or surger* or surgic*).ab,ti,kw. 2629595

7 1 or 2 or 3 367649

8 4 or 5 or 6 2749003

9 7 and 8 23842

10 (playlist* or spotify or mp3 or ipod or music* or acoustic stimul* or auditory stimul* or song* or melod*).ab,ti,kw. 47964

11 1 or 2 or 10 88189

12 occupational stress/ or burnout, professional/ 15989

13 stress, psychological/ or burnout, psychological/ 127646

14 Stress, Physiological/ 79605

15 (stress* or perform* or demand* or focus* or anxiet* or relax* or work?load or task?load or heart rate or blood pressure or skin conduct* or STAI* or SURG?TLX*).ab,ti,kw. 6313464

16 12 or 13 or 14 or 15 6368917

17 Operating Rooms/ 14724

18 Surgical Procedures, Operative/ 56237

19 8 or 17 or 18 2759722

20 11 and 16 and 19 1708

21 limit 20 to (english language and humans) 1072

Cochrane Database

Search Name:

Date Run: 12/08/2021 17:16:57

Comment:

ID Search Hits

#1 MeSH descriptor: [Music] explode all trees 673

#2 MeSH descriptor: [General Surgery] explode all trees 359

#3 MeSH descriptor: [Surgeons] explode all trees 165

#4 MeSH descriptor: [Operating Rooms] explode all trees 229

#5 MeSH descriptor: [Music Therapy] explode all trees 875

#6 MeSH descriptor: [Anesthesia] explode all trees 19853

#7 MeSH descriptor: [Acoustic Stimulation] explode all trees 1113

#8 MeSH descriptor: [Surgical Procedures, Operative] explode all trees 122866

#9 playlist* or spotify or mp3 or ipod or music* or acoustic stimul* or auditory stimul* or song* or melod*:ab,ti,kw 17858

#10 #1 OR #5 OR #7 OR #9 17858

#11 ((operating or operation) NEAR/3 (room* or staff or theatre*)) or surgeon* or an?esthe* or pre?operat* or post?operat* or peri?operat* or intra?operat* or surger* or surgic*:ab,ti,kw 347722

#12 #2 OR #3 OR #4 OR #8 OR #11 377653

#13 stress* or perform* or demand* or focus* or anxiet* or relax* or work?load or task?load or heart rate or blood pressure or skin conduct* or STAI* or SURG?TLX*:ab,ti,kw 586034

#14 MeSH descriptor: [Occupational Stress] explode all trees 409

#15 MeSH descriptor: [Stress, Psychological] explode all trees 6436

#16 MeSH descriptor: [Stress, Physiological] explode all trees 4558

#17 MeSH descriptor: [Psychological Distress] explode all trees 161

#18 MeSH descriptor: [Psychological Tests] explode all trees 22478

#19 #13 OR #14 OR #15 OR #16 OR #17 OR #18 597122

#20 #10 AND #12 AND #19 2382
